# Supplementary material for: Exercise Adherence in Men with Prostate Cancer Undergoing Androgen Deprivation Therapy: A Systematic Review and Meta-Analysis
Source: Cancers (Basel). 2022 May 16;14(10):2452. doi: 10.3390/cancers14102452 (PMC9139246; doi:10.3390/cancers14102452)
Supplement: Supplementary file 1 [file cancers-14-02452-s001.zip › cancers-1714610-supplementary.pdf]

Supplementary Table S1. Results of subgroup analyses for meta-analyses on the effects of exercise on quality of life, aerobic fitness, fatigue, and upper- and lower-body strength.

Outcome	*n* = studies	*n* = participants	*p* value	SMD [95% CI]
**Quality of life**	**8**	**493**	**0.11**	**0.15 [-0.03, 0.32]**
Adherence				
>75%	7	399	0.08	0.18 [-0.02, 0.37]
≤75%	1	94	0.91	0.02 [-0.38, 0.43]
Not reported	0	0	-	Not estimable
Exercise mode				
Aerobic	1	94	0.91	0.02 [-0.38, 0.43]
Resistance	2	83	0.83	0.05 [-0.38, 0.48]
Mixed mode	5	316	0.08	0.21 [-0.02, 0.44]
Other	0	0	-	Not estimable
Length				
≤ 12 weeks	4	216	0.24	0.19 [-0.13, 0.51]
> 12 weeks	4	277	0.34	0.12 [-0.12, 0.35]
**Aerobic fitness**	**15**	**939**	**<0.01**	**0.50 [0.15, 0.85]**
Adherence				
>75%	11	683	0.07	0.42 [-0.03, 0.87]
≤75%	2	191	<0.01	0.75 [0.21, 1.28]
Not reported	2	65	0.26	0.77 [-0.57, 2.11]
Exercise mode				
Aerobic	2	136	0.17	0.61 [-0.26, 1.47]
Resistance	3	151	0.18	0.48 [-0.22, 1.17]
Mixed mode	9	595	0.08	0.49 [-0.05, 1.02]
Other	1	57	0.12	0.42 [-0.11, 0.94]
Length				
≤ 12 weeks	8	455	0.06	0.58 [-0.02, 1.18]
> 12 weeks	7	484	0.06	0.39 [-0.02, 0.80]
**Fatigue**	**7**	**511**	**0.44**	**-0.09 [-0.33, 0.15]**
Adherence				
>75%	6	417	0.20	-0.16 [-0.41, 0.08]
≤75%	1	94	0.19	0.27 [-0.14, 0.68]
Not reported	0	0	-	Not estimable
Exercise mode				
Aerobic	0	0	-	Not estimable
Resistance	1	58	0.89	-0.04 [-0.55, 0.48]
Mixed mode	6	453	0.44	-0.11 [-0.39, 0.17]
Other	0	0	-	Not estimable
Length				
≤ 12 weeks	4	259	0.02	-0.29 [-0.54, -0.05]
> 12 weeks	3	252	0.15	0.18 [-0.07, 0.43]
**Upper-body strength**	**8**	**307**	**0.03**	**0.34 [0.04, 0.63]**
Adherence				
>75%	5	260	0.11	0.26 [-0.06, 0.58]
≤75%	0	0	-	Not estimable

Not reported	3	47	0.08	0.65 [-0.07, 1.37]
Exercise mode				
Aerobic	0	0	-	Not estimable
Resistance	2	81	<0.01	0.72 [0.27, 1.18]
Mixed mode	6	226	0.24	0.17 [-0.11, 0.45]
Other	0	0	-	Not estimable
Length				
≤ 12 weeks	6	226	0.24	0.17 [-0.11, 0.45]
> 12 weeks	2	81	<0.01	0.72 [0.27, 1.18]
**Lower-body strength**	**8**	**406**	**<0.01**	**0.54 [0.26, 0.82]**
Adherence				
>75%	7	383	<0.01	0.57 [0.27, 0.87]
≤75%	0	0	-	Not estimable
Not reported	1	23	0.66	0.19 [-0.67, 1.05]
Exercise mode				
Aerobic	0	0	-	Not estimable
Resistance	3	151	<0.01	0.70 [0.21, 1.19]
Mixed mode	4	198	<0.01	0.59 [0.30, 0.87]
Other	1	57	0.64	-0.12 [-0.64, 0.40]
Length				
≤ 12 weeks	5	255	0.01	0.45 [0.11, 0.79]
> 12 weeks	3	151	<0.01	0.70 [0.21, 1.19]
